# Retrospective Change-Points Detection for Multidimensional Time Series of Arbitrary Nature: Model-Free Technology Based on the *ϵ*-Complexity Theory

**DOI:** 10.3390/e23121626

**Published:** 2021-12-02

**Authors:** Alexandra Piryatinska, Boris Darkhovsky

**Affiliations:** 1 Department of Mathematics, San Francisco State University, 1600 Holloway Ave., San Francisco, CA 94132, USA; 2 Institute for Systems Analysis, FRC CSC RAS 9 Pr. 60-Letiya Oktyabrya, 117312 Moscow, Russia; darbor2004@mail.ru

**Keywords:** *ϵ*-complexity, change-point detection, model-free segmentation

## Abstract

We consider a retrospective change-point detection problem for multidimensional time series of arbitrary nature (in particular, panel data). Change-points are the moments at which the changes in generating mechanism occur. Our method is based on the new theory of ϵ-complexity of individual continuous vector functions and is model-free. We present simulation results confirming the effectiveness of the method.

## 1. Introduction

In retrospective studies, all observations are collected a priori. A retrospective analysis of multivariate time series begins by checking their homogeneity. We call data homogeneous if the same mechanism generates them. When the homogeneity assumption is violated (i.e., the data generation mechanism changes during their collection), we must perform segmentation of the data into homogeneous increments. In cases of *stochastic* data generating mechanism, the segmentation problem is well-known as the “change-point detection” problem in the retrospective setting. A vast amount of literature is devoted to the change-point detection for stochastic processes in both “off-line” and “on-line” formulation, see e.g., [1,2,3].) In this case, the change-points are the moments of changes in their probabilistic characteristics.

Segmentation problems arise in econometrics and financial mathematics. In these areas, the change-points are called structural breaks. In the last 20 years, detecting structural breaks in the so-called panel data has attracted the attention of many researchers. Panel data is data that contains observations about different cross-sections across time. Groups that may make up panel data series include countries, firms, individuals, or demographic groups. The primary difference between panel data models and time series models is that panel data models allow for heterogeneity across groups and introduce individual-specific effects. Panel data are usually high-dimensional (have hundreds of components). In the literature, there are many interesting publications on such problems (see e.g., [4,5,6,7]). In such studies, only the stochastic models were used to model panel data.

However, in many applications, the data are more complex and cannot always be modeled as stochastic processes. There is a large class of complex systems that, being *deterministic, exhibit stochastic behavior*. Such systems are called chaotic. The existing mathematical theory of chaotic systems (see, for example, [8]) suggests that they should be described by an *unchanging equation of evolution*. Meanwhile, in real chaotic systems, changes in parameters can occur, resulting in conditions in which the system can pass from one regime to another. This is how the *multifractality* phenomenon can arise, which is currently receiving much attention in the literature (see, for example, [9]). It follows that the problem of checking homogeneity and segmentation is no less critical for the analysis of chaotic systems.

These considerations lead to a broader interpretation of the term “change-point” (or “moments of disorder”). Namely, we mean by this term *the moment of change in the generating mechanism of a multidimensional time series, regardless of what its nature is*.

All currently known methods for solving problems on the change-point detection of *stochastic processes* in one way or another rely on their models (i.e., on the knowledge about the model that generates data). However, information about data generation mechanisms is not always available. A typical example here is the EEG signal, which, according to most experts, is one of the most demanding physical processes to study, and there is no generally accepted model of such a process. The situation is similar in this sense in applications to financial series, some biological problems, etc., where there are no firmly established models of the observed processes. The situation with chaotic systems seems to be even more complicated in this respect. Thus, in many applications, a *vicious circle* situation arises: for adequate data segmentation, it is required to know the model of this data, and the model can be built only after data segmentation into “homogeneous” fragments.

In this paper, we propose a *model-free method* for retrospective detection of multiple change-points in multidimensional time series. This method is based on the theory of the ϵ-complexity of continuous vector-functions. The theory of the ϵ-complexity of continuous functions was developed in our recent works [10,11]. It enables us to develop a model-free method for detection of change-points (i.e., in our terminology, *moments of change in the generating mechanism*) for multivariate time series of arbitrary nature (stochastic, deterministic, or mixed). We demonstrate the effectiveness of the method with the help of simulations. In our simulations, we consider examples of multivariate time series, which are generated by a multidimensional stochastic process with dependent components (vector autoregressive model); multidimensional chaotic deterministic processes, with some dependent components; and the mixed process, which has some stochastic components and some chaotic deterministic components.

The proposed method is a modification of the method published in [12]. In that paper, we presented the method for retrospective detection of change-points in a time series of small dimensions (6 independent components of a vector series). The main differences between this article and [12] are as follows:

(a) Here, we rely on more exact definitions and formulations of the theory of ϵ-complexity (which did not lead to a change in the basic relations), given in [11]. (The general idea of ϵ-complexity was created about 8 years ago. This idea was quickly implemented in computer algorithms and successfully used to solve problems of disorder detection and classification. However, as is often the case with new ideas, rigorous mathematical formulations took more time. The definitions and results of the theory are given in [11] and, in this paper, we rely on the improved definitions and theory.)
(b) We made a change in the algorithm for calculating the complexity coefficients (see Section 2 below), which made it possible to detect changes in mean value and variance.
(c) For simulations of stochastic components, we used a general multidimensional linear model of high dimension (50 components of the vector series, interconnected by linear relations) and investigated the possibility of detecting changes in the matrices of this model and the mean value of the vector series.
(d) We investigated the effectiveness of our method in the case when abrupt changes did not occur in all components of the time series.

The paper is organized as follows: in Section 2, we present the basic concepts and results of the theory of ϵ-complexity at a meaningful level, referring the reader to the exact formulations in [11]. Section 3 describes the method of retrospective detection of changes in the mean value, variance, and parameters of chaotic processes in multidimensional time series of an arbitrary nature. Section 4 shows the results of the simulations. Section 5 provides conclusions.

## 2. Brief Description of the Results of the Theory of ϵ-Complexity

In this section, we present the results of the theory of the ϵ-complexity of continuous vector functions at the meaningful level. Mathematically rigorous definitions and results are given in [11].

Let a continuous vector function $x\left(\cdot \right)=\left(x_{1}\left(\cdot \right),\ldots ,x_{m}\left(\cdot \right)\right)$ be defined on a finite time interval. Denote $R_{i}=\max_{t}\left|x_{i}\left(t\right)\right|,\;i\in I=\left(1,\ldots ,m\right)$ and we will assume that $R=\min_{i\in I}R_{i}>0$.

Without loss of generality, we assume that vector function x(·) is defined on [0,1]. Consider a uniform grid on [0,1] with some step 1>h>0. We call an arbitrary Borel function that transfers a finite set of discrete vector function values (i.e., *m*-dimensional vectors, the number of which is determined by the value *h*) into some bounded vector function on [0,1] *the method of recovery (approximation) of a continuous vector function* (a uniform metric is introduced in the space of bounded vector functions).

Let us fix an arbitrary countable set of Borel vector functions with values in the space of bounded vector functions depending, respectively, on 1,2,3,… arguments. We call a *list* the union of these countable sets. The list contains a countable set of recovery methods for all h>0.

Let us fix some list F of recovery methods. Throughout what follows, the symbol F denotes an arbitrary nonempty subset of F containing some collection of Borel vector functions from 1,2,3,…… arguments.

The sets F (and, accordingly, the lists F for F=F) are *admissible* if they contain methods of approximation by piecewise constant (stepwise) vector functions and power polynomials.

The recovery methods are “physically realizable” if they can be represented as computer programs. Such recovery methods contain a finite set of bounded piecewise continuous vector functions of a finite number of variables with values in the space of bounded vector functions. Note that any finite set of “physically realizable” recovery methods is included in some admissible list.

We set

$$\delta_{i}^{F}\left(h\right)=\underset{{\hat{x}}_{i,h}\left(\cdot \right)\in F}{\inf}\underset{t\in [0,1]}{\sup}\left|{\hat{x}}_{i,h}\left(t\right)-x_{i}\left(t\right)\right|,\;i=1,2\ldots m.$$

Here, the symbol ${\hat{x}}_{i,h}\left(\cdot \right)\in F$ denotes the estimates of the *i*-th component of the vector function x(·) by its finite set of values with step *h* obtained by methods of the family F. In the case when F=F, all functions included in F are used for evaluation.

**Lemma 1**  (Density lemma)**.** *Let F be an arbitrary fixed admissible list. The set of continuous vector functions that cannot be precisely reconstructed from a finite number of functions’ values by the methods from the list F is everywhere dense in the space of all continuous vector functions.*

Vector functions that *cannot be exactly reconstructed* by methods of an arbitrary nonempty admissible subset F⊆F we call *F-nontrivial*.

Let F be a fixed admissible list and F⊆F be an arbitrary nonempty admissible subset. Let x(t) be F-nontrivial vector function. For sufficiently small ϵ>0, put

$$h_{i}^{*}\left(\epsilon ,F\right)=\left\{\begin{array}{cc} \inf\{h\leq 1:\frac{\delta_{i}^{F}\left(h\right)}{R_{i}}>\epsilon \}, & \mathrm{if}\;x_{i}\left(\cdot \right)\;is\;F-\mathrm{nontrivial}\\ 1, & \mathrm{in}\;\mathrm{opposite}\;\mathrm{case} \end{array}\right.$$

Set

$$h_{x}^{*}\left(\epsilon ,F\right)=\prod_{i=1}^{m}h_{i}^{*}\left(\epsilon ,F\right)$$

**Definition** **1.** The (ϵ,F)-complexity of a continuous vector function x(·) is the value $S_{x}\left(\epsilon ,F\right)=-\log h_{x}^{*}\left(\epsilon ,F\right)$.

If a vector function is not F-nontrivial (i.e., it can be reconstructed exactly from a finite number of its values), then we assume that its (ϵ,F)-complexity is zero (see definition above). Thus, the Density Lemma implies that “almost all” continuous vector functions have nonzero (ϵ,F)-complexity for any F⊆F for an arbitrary fixed admissible list F.

Note that $h_{i}^{*}\left(\epsilon ,F\right)>0$ for ϵ>0 and $\lim_{\epsilon \to 0}h_{i}^{*}\left(\epsilon ,F\right)=0$ if $x_{i}$ is F-nontrivial. On the other hand, $\lim_{h\to 0}\max_{i}\delta_{i}^{F}\left(h\right)=0$. Therefore, for any (sufficiently small) ϵ>0, there exists η(ϵ)>0,η(ϵ)→0 for ϵ→0 such that $\max_{i}\delta_{i}^{F}\left(h_{x}^{*}\left(\cdot \right)\right)\leq \eta \left(\epsilon \right)$.

Considering that $1/h_{x}^{*}\left(\epsilon ,F\right)$ is an estimate of the number of values of a vector function, we obtain that the (ϵ,F)-complexity is (logarithm) of the number of its values that are required for its reconstruction by methods of the family F with a relative error of at most $R^{-1}\eta \left(\epsilon \right)$. In other words, we can say that this is the *the shortest description of the vector function* by these methods with a given precision. In this sense, our definition is consistent with the main idea of A.N. Kolmogorov that the complexity of an object should be measured by the length of its shortest description.

In most modern applications, a researcher deals with time series given by a discrete set of their values on a uniform grid. Assuming that such a collection of values is the *restriction of a continuous vector function on some uniform grid*, we can extend the theory of ϵ-complexity to this case.

Let the number 0<S<1 be chosen. Let us discard some part of the initial *n* values of the vector function so that after discarding [Sn], values will retain (discarding the sample points should be done in such a way that the remaining sample points are approximately evenly spaced). Thus, *S* is the fraction (of the total *n*) of sample points that remain after discarding.

Denote by $\epsilon_{i}\left(n,F,S\right)\overset{\mathrm{def}}{=}\epsilon_{i}\left(\cdot \right)$*minimal* (by all methods of the collection F) recovery error for the *i*-th components of the vector function x(·) (now it is a multidimensional vector time series) by the remaining [Sn] time points. The recovery error can be measured in any finite dimensional standard norm.

We set

(1) $$\log\rho =\sum_{i}\left(\log\frac{\epsilon_{i}}{R_{i}}+\log\epsilon_{i}\right)\;$$

Let us present the main result of the theory of ϵ-complexity for the case when a continuous vector function is given by its restriction on a fixed uniform grid.

*For any Hölder vector function from an everywhere dense set, given by its restriction on a fixed uniform grid, the following relation holds*

(2) logρ≈A(n)+B(n)logS.

*The richer set of approximation methods F, and the greater the number of function values n on a fixed time interval, the more accurate the recovery is.* (In our paper [13], relation (2) was given for the case when the sum in relationship (1) contained only the first term. However, the general theory of ϵ-complexity implies that the addition of the second term in (1) does not fundamentally change relation (2). The need to introduce the second term in (1) is caused by the desire to capture changes in *the mean and the variance*. Without this term, such changes may not be detected using the complexity coefficients).

The coefficients A(n),B(n) in (2) will be called the *ϵ-complexity coefficients*. The complexity coefficients have nothing to do with the time series generation mechanism (i.e., the model that generates them). Therefore, any method that utilizes these coefficients will be automatically model-free. *The method for detecting change-points in a multidimensional time series of an arbitrary nature, described below, is based on the ϵ-complexity coefficients*.

## 3. Method for Detection of Changes in Generating Mechanism in Multidimensional Time Series of Arbitrary Nature

The main idea of our methodology for retrospective detection of change-points in multidimensional time series of arbitrary nature is as follows.

Let $X={\left\{x\left(t\right)\right\}}_{t=1}^{N}$ be a time series with unknown moments of change in the generation mechanism (MCGM) $t_{i},i=2,\ldots ,k$ (such moments may not be present). We emphasize that the mechanisms for generating the series *are unknown and can be stochastic, deterministic, or mixed*.

Segments of the series $\left[t_{i},t_{i+1}\right],\;\;t_{1}=1,\;t_{k+1}=N$, which are generated by the same mechanism, we call *homogeneous* and assume that the homogeneity segments are sufficiently long.

As shown in Section 2, the ϵ-complexity of a segment is determined by the parameters $R=\left(A,B\right)$. Notice that in relationship (2), *A* and *B* depend on *n*; further, the windows size *n* will be fixed, and therefore, A(n)=A and B(n)=B.

Let us choose a window of size *n* (it is assumed that $n\ll \min_{i}\left(t_{i+1}-t_{i}\right)$) and for each segment of the series x(t),t∈[jn+1,(j+1)n],j=0,1…,[N/n], we will calculate the complexity coefficients R(j+1). As a result, we obtain a new *diagnostic* vector sequence ${\left\{R\left(j\right)\right\}}_{j=1}^{j=[N/n]}$.

The key idea of the proposed method is the following *hypothesis*: on the *i* -th homogeneity segment $\left[t_{i},t_{i+1}\right]$ of the time series *X* for $t_{i}\leq t,\;\left(t+n\right)<t_{i+1}$ (and for corresponding intervals of the diagnostic sequence), the complexity coefficients satisfy the relation

$$R\left(j\right)=R_{i}+\xi^{i}\left(j\right),$$

where $\xi^{i}\left(j\right)$ is a random vector sequence with zero mathematical expectation.

Note that when the moving window crosses any moment of the MCGM (if our hypothesis is true), the mathematical expectation of the sequence R changes according to some transient process from one constant to another. However, since, by assumption, the window size is significantly less than the length of any homogeneity segment, such a transient will not significantly affect the estimates of the MCGM.

Thus, if the given hypothesis is valid, the problem of time series segmentation is reduced to the change-point detection problem with the change in the mean values in the diagnostic vector sequence R(j).

To detect change-points in diagnostic sequences, we use a 3-step procedure (introduced in [2]) based on the family of statistics

$$Y\left(s,\delta \right)={\left(\left(N-s\right)s/N^{2}\right)}^{\delta }\left(s^{-1}\sum_{k=1}^{s}z\left(k\right)-{\left(N-s\right)}^{-1}\sum_{k=n+1}^{N}z\left(k\right)\right),$$

where $0\leq \delta \leq 1,1\leq s\leq N-1,N=\left[N/n\right],\;Z={\left\{z\left(k\right)\right\}}_{k=1}^{N}$—implementation of the components of the diagnostic sequence R(j).

The first version of this family of statistics was proposed in [14]; a short description of the 3-step detection procedure can be found in [13].

It can be shown (see [2] for details) that under broad assumptions about random sequences $\left\{\xi^{i}\left(j\right)\right\}$, the statistic leads to asymptotically (for N→∞) minimax estimates for the moment of change in the generation mechanism.

So, our method for detecting MCGM in a multidimensional time series is as follows:

1. Choose the size of the disjoint intervals or sliding window for the considered time series.
2. Calculate complexity coefficients for each window. For this purpose, the parameter *S* in (2) is assigned different values $S_{1},\ldots ,S_{k}$; for each value of $S_{j},j=1,\ldots k$, the value $\log\rho_{j}$ is determined (in this case, averaging over all possible locations of the row counts remaining after discarding) and then using the set of pairs $\left(\log\rho_{j},\log S_{j}\right)$, the complexity coefficients A,B for the window under consideration are calculated using the standard least squares method. The scheme of these calculations is described in detail in [12]. It is necessary to take into account the replacement of the error appearing there by the value ρ from (1).
3. The above 3-step change-point detection procedure is applied to each component of the sequence of complexity coefficients.
4. We combine detected change-points from both components of the complexity coefficients sequence. As a result, we obtain the estimates of MCGM.

## 4. Simulations

In this section, we present our simulations, which demonstrate the performance of our method.

### 4.1. Stochastic and Deterministic Processes Used in the Simulations

Lets us first describe the processes that we employed in our simulation study.

The *stochastic process* we utilize here is vector autoregressive process of order *p* (denoted by VAR(p)). It is given as follows.

(3) $$x_{t}=\mu +\Theta_{1}x_{t-1}+\cdots +\Theta_{p}x_{t-p}+u_{t},t=0,\pm 1,\pm 2,\ldots ,\;$$

where $x_{t}$, $u_{t}$, and μ are (K×1) vectors and $\Theta_{i}$ are (K×K) matrices for each i=1,…,p. In addition, the error term $u_{t}$ is a white noise random vector such that $E(u_{t})=0$, $E\left(u_{t}u_{t}^{\prime }\right)=\Sigma_{u}$, and $E(u_{t}u_{s}^{\prime })=0$ for s≠t, where $\Sigma_{u}$ is a (K×K) positive definite matrix. Such model is often used to simulate panel data and investigate structural breaks in panel data, see e.g., [15]. This model can be rewritten in a compact form (see e.g., [16]),

(4) $$X_{t}=\mu +\Theta X_{t-1}+U_{t},\;t=0,1,2,\ldots \;$$

where $X_{t}={\left[x_{t}^{{}^{\prime }},x_{t-1}^{{}^{\prime }},\ldots x_{t-p+1}^{{}^{\prime }}\right]}^{\prime }$, $\mu ={\left[\mu^{\prime },0,\ldots ,0\right]}^{\prime }$, $U_{t}={\left[u_{t}^{{}^{\prime }},0^{{}^{\prime }},\ldots 0^{{}^{\prime }}\right]}^{\prime }$ are (Kp×1) vectors and

(5) $$\Theta =\left[\begin{array}{ccccc} \Theta_{1} & \Theta_{2} & \ldots & \Theta_{p-1} & \Theta_{p}\\ I_{k} & 0 & \ldots & 0 & 0\\ 0 & I_{k} & \ldots & 0 & 0\\ \vdots & & \ddots & \vdots & \vdots \\ 0 & 0 & \ldots & I_{k} & 0 \end{array}\right]\;$$

is a (Kp×Kp) matrix. The model (5) is stable if and only if $|\lambda_{\max}\left(\Theta \right)|<1$, where $|\lambda_{\max}\left(\Theta \right)|$ denotes the largest absolute value of the eigenvalues of the matrix Θ.

Using this model, we simulate multivariate time series with dependent components. Our segments of multivariate time series have either different variance covariance matrices $\Sigma_{u_{1}}$ and $\Sigma_{u_{2}}$ or different lag matrices $\Theta_{1}$ and $\Theta_{2}$. The mean values of the components of our processes change too. In our simulations, we report spectral norms of the matrices $\Sigma_{u_{i}}$, $\Theta_{i}$, i=1,2.

Let us remind that the spectral norm of a matrix *D* is the largest singular value of the matrix *D*, i.e., the square root of the largest eigenvalue of the matrix $D^{*}D$, where $D^{*}$ denotes the conjugate transpose of *D*:

$${∥D∥}_{2}=\sqrt{\lambda_{\max}\left(D^{*}D\right)}$$

(see e.g., [17]).

We also consider chaotic deterministic processes in discrete time. The change in generating mechanisms in some of these processes will correspond to the change in the parameters. In another case, we concatenate different chaotic processes, where changes are the points of the concatenation.

All processes that we consider are as follows: $x_{t}=f\left(x_{t-1}\right)$, t=1,2…. The functions f(x) are described below.

We consider the following maps.

1. The logistic map
  (6) $$f\left(x\right)=\alpha x\left(1-x\right)\;x_{0}\in \left(0,1\right)\;$$
  The parameter for this process is α. In our simulations, we use 3.85≤α≤4. It is well known (see [18]) that under these parameter values, the corresponding processes exhibit chaotic behavior.
2. The quadratic map, see e.g., [19]
  (7) $$f\left(x\right)=c-x^{2},\;0<c\leq 2,\;x_{0}\in \left(0,1\right).$$
3. Process 3
  (8) $$f\left(x\right)=1-|1-2x|,\;x_{0}\in \left(0,1\right)$$
4. The Interval map, see e.g., [20]
  (9) $$f\left(x\right)=2x\left(mod\;1\right),\;x_{0}\in \left(0,1\right).$$

Let us notice that the process 3 given by function (8) and the Interval map given by function (9) does not have parameters that can be changed.

We also consider two-dimensional maps of the following form $z_{t}=f\left(z_{t-1}\right)$, where

$$z_{t}=\left(\begin{array}{c} x_{t}\\ y_{t} \end{array}\right).$$

5. The Hénon Map, see [21]
  (10) $$f\left(z\right)=\left(\begin{array}{c} 1-ax^{2}+y\\ bx \end{array}\right)\;$$
6. The Ikeda map, see [22]
  (11) $$f\left(z\right)=\left(\begin{array}{c} 1+\mu (x\cos\phi (x,y)-y\sin\phi (x,y)\\ \mu (x\sin\phi (x,y)+y\cos\phi (x,y)) \end{array}\right)\;$$
  Here, $\phi \left(x,y\right)=0.4-\frac{6}{1+x^{2}+y^{2}}$

### 4.2. Results of Simulations

In each example, we simulate multidimensional time series. We take into account the fact that in chosen processes, stationary probability distributions are established sufficiently fast. Here, we discard the beginning of the simulated process before such stabilization. We will concatenate three or four homogeneous multidimensional time series. The length of each homogeneous component will be 5000. In some examples, we will change the coefficients in the models. In other examples, we will link different deterministic processes. After concatenation, we will separate each multidimensional time series into non-overlapping segments of length 100. For each segment, the ϵ-complexity coefficients will be calculated. As a result, we generate two-dimensional diagnostic sequences. For each component of a diagnostic sequence, we will apply the 3-step nonparametric change-point detection procedure of Brodsky and Darkhovsky. If we observe a change in at least one component of the diagnostic sequence, we will assume that the change occurred. To ensure the stability of the results, we perform 1000 replications of each numerical experiment.

**Example** **1.** *Stochastic process, VAR(1).*

In this example, we consider the VAR(1) process. We choose K=50; as a result, we have 50 dimensional multivariate time series with dependent components. We simulated 5 different segments of length 5000, concatenated them, and obtained the time series of length 25,000 with four change-points (or MCGM). We performed 1000 replications of the experiment.

The description of the segments is provided in Table 1. The first column lists the type of matrices that define the model. The 2nd, 3rd, 4th, and 5th columns correspond to the specification for each segment. In the first row, one can see which model matrices are the same and which are different for corresponding segments. In the second row, we provided the corresponding norms of the model matrices. In the third row, one can see which variance–covariance matrices are the same and which are different for corresponding segments. In the fourth row, we provided corresponding norms for variance–covariance matrices. The first MCGM corresponds to the change in the mean in half of the components. The second disorder corresponds to the change in model matrix Θ. The third MCGM corresponds to the change in variance–covariance matrix *U*. The fourth disorder is the change in the mean for all components.

**Figure 1:**
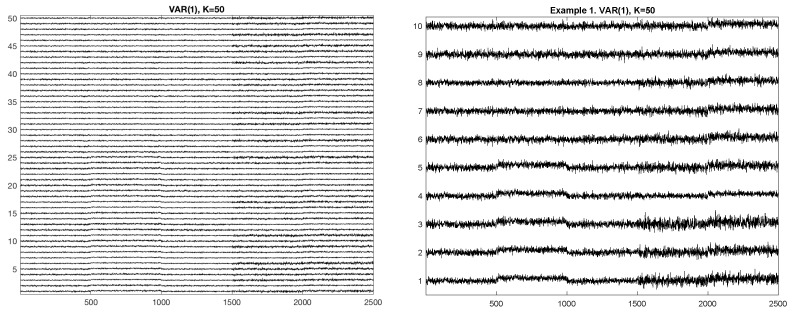
Example 1. Realization of the VAR(1) process. **Left**: All 50 components. **Right**: selected 10 components.

Figure 1 shows an example of the simulated process from Example 1. The Left plot shows all 50 components of the process. In this realization, each homogeneous segment (the one with the same generating mechanism) has a length of 500 points. The right plot shows only ten components. It allows us to see better the behavior of the process.

**Figure 2:**
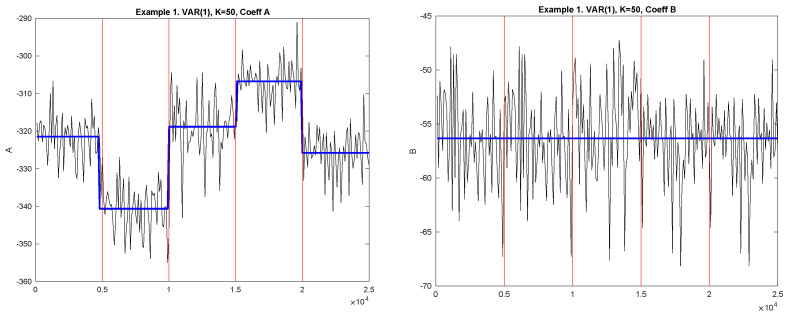
Example 1. Diagnostic sequences and detected MCGM. **Left**: Coefficient A; **Right**: Coefficient B. Black solid lines correspond to the diagnostic sequences; horizontal blue lines correspond to the mean values between the detected MCGM. The jump points correspond to the detected change-points. The vertical red lines correspond to the true change-points.

Figure 2 shows the examples of the diagnostic sequences *A* (Left plot) and *B* (Right plot) and detected change-points. Black solid lines correspond to the diagnostic sequences, horizontal blue lines correspond to the mean values between the detected change-points. The jump points correspond to the detected change-points. The vertical red lines correspond to the true change-points.

The numerical results are presented in Table 2 and Table 3. The percentage of the number of detected points for diagnostic sequences of coefficients *A* and *B* are presented in Table 2. Let us notice that the coefficient *B* was not useful for detecting change-points in this example. The percentages of correctly found numbers of each of the four change-points (true positive rate) and corresponding bootstrap confidence intervals are presented in Table 3. To compare the new method with the old method, we present in Table 4 the percentages of correctly found numbers of each of the four change-points (true positive rate) and corresponding bootstrap confidence intervals for our old method.

In the first change-points, the change occurred in 50% of the components, and the size of the shift was approximately 0.77σ=2. Here, σ is the maximal standard deviation of the components. If we reduce the number of components or decrease the size of the shift, our accuracy will decrease. For the last change-point, we decrease the size of the change in the mean but have a change in the mean of all components. In this case, the shift was approximately 0.75σ.

To measure differences between the matrices and variance–covariance matrices for which we were able to detect changes, we report spectral norms $∥\Theta_{1}-\Theta_{2}{∥}_{2}$ and $∥\Sigma_{u_{1}}-\Sigma_{u_{2}}{∥}_{2}$ and ratios $∥\Theta_{1}-\Theta_{2}{∥}_{2}/(0.5∥\Theta_{1}{∥}_{2}+0.5∥\Theta_{2}{∥}_{2})$, $∥\Sigma_{u_{1}}-\Sigma_{u_{2}}{∥}_{2}/(0.5∥\Sigma_{u_{1}}{∥}_{2}+0.5∥\Sigma_{u_{2}}{∥}_{2})$. Thus, for the second change-point, when the change occurred in the model matrix, $∥\Theta_{1}-\Theta_{2}{∥}_{2}=0.053$ and $∥\Theta_{1}-\Theta_{2}{∥}_{2}/(0.5∥\Theta_{1}{∥}_{2}+0.5∥\Theta_{2}{∥}_{2})=0.49$. For the third change-point, where change occurred in variance–covariance matrix, $∥\Sigma_{u_{1}}-\Sigma_{u_{2}}{∥}_{2}=12.64$ and $∥\Sigma_{u_{1}}-\Sigma_{u_{2}}{∥}_{2}/(0.5∥\Sigma_{u_{1}}{∥}_{2}+0.5∥\Sigma_{u_{2}}{∥}_{2})=0.25$.

As one can see from Table 3 and Table 4, we detected the first and third change-points with the proposed method, and the old approach did not detect them. In the case of the fourth change-point, the old method detected 56% of simulations while the proposed method detected 78.3% of simulations.

**Example** **2.** *Chaotic deterministic processes.*

In this example, we created a seven-dimensional series with chaotic components. The processes and parameters for each process are presented in Table 5. In the first column, we gave the index of the component. In the second column, we presented the name of the process. In parentheses, we provided the reference to the equations that generate the process. For components 1–6 in columns 3, 4, and 5 (with titles Segment 1, Segment 2, Segment 3), we provided parameters of the processes used to generate corresponding segments. For component 7, the processes do not have parameters, and we provide the reference to the generating equation and its name. We generated segment 4 the same way as segment 3, but we added a shift of size 0.5 of the standard deviation of the components of segment 3.

One can see that the first change in generating mechanism occurred in all components. The second change occurred in the parameters of the first six components. The third change in generating mechanism is due to the shift.

**Figure 3:**
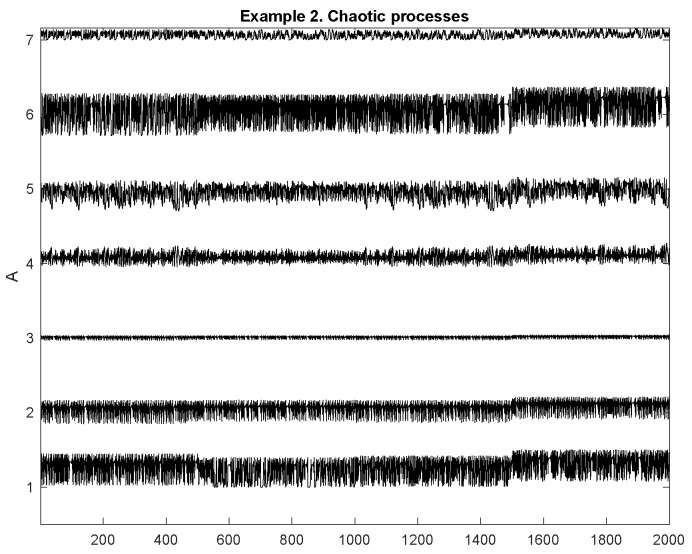
Example 2. Realization of the chaotic multidimensional process.

Figure 3 shows an example of the simulated process from Example 2. In this realization, each homogeneous segment has length 500 points.

**Figure 4:**
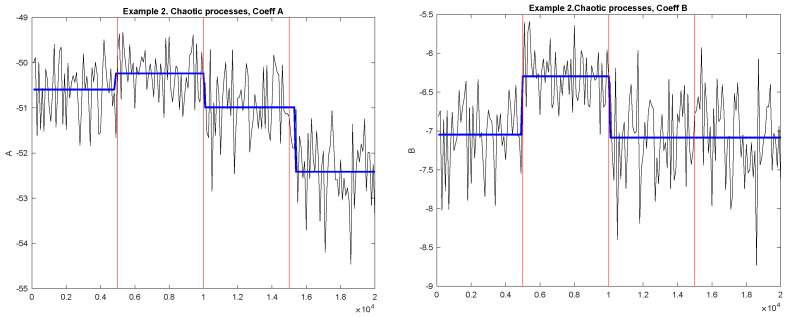
Example 2. Diagnostic sequences and detected MCGM. **Left**: Coefficient A; **Right:** Coefficient B. Black solid lines correspond to the diagnostic sequences; horizontal blue lines correspond to the mean values between the detected change-points. The jump points correspond to the detected change-points. The vertical red lines correspond to the true change-points.

Figure 4 shows the examples of the diagnostic sequences *A* (Left plot) and *B* (Right plot) and detected change-points for Example 2. Black solid lines correspond to the diagnostic sequences; horizontal blue lines correspond to the mean values between the detected change-points. The jump points correspond to the detected change-points. The vertical red lines correspond to the actual change-points.

The numerical results are presented in Table 6 and Table 7. The percentage of the number of detected points for diagnostic sequences of coefficients *A* and *B* are presented in Table 6. The percentages of correctly found numbers of each of the three change-points (true positive rate) and corresponding bootstrap confidence intervals are presented in Table 7. The results for the old method are presented in Table 8. Let us notice that the coefficient *B* was more efficient for detecting changes in generating mechanism when one chaotic process changes by another one. In this case, we do have change in the Hölder constant and, therefore, coefficient *B* detection works best. Let us notice that the second MCGM was detected only in 75% of cases by coefficient *B*. In this case, it was no change for one of the components.

We observe that the first two points can be detected using coefficient *B*. It agrees with our hypothesis that for such processes, the Hölder constant changes. The shift cannot be detected using coefficient *B*. In terms of coefficient *B*, our proposed method and our old method detect a similar proportion of first and second change-points. However, we could not detect a change in the mean of the multivariate process using the old method (see, Table 8). The new method detected this change in 95.3% of the simulations.

**Example** **3.** *Mixed process.*

In this example, we combine processes from the first examples and parametric processes from the second example. As a result, we obtained a multidimensional time series that has stochastic and deterministic components. In this example, we simulated 20 components of the multivariate stochastic process and eight components of deterministic processes. We simulated five homogeneous segments of length 5000. The total length is 25,000. There are four MCGMs.

The processes and parameters for each process are presented in Table 9.

In the first column, we present the index of the component. In the second column, we provide the name of the process. In parentheses, we provide the reference to the equations that generate the process. The first 20 components are trajectories of the VAR(1) model. In Table 9, one can see which matrices are the same and which are different for different segments. For components 21–28 in columns 3, 4, 5, and 6 (with titles Segment 1, Segment 2, Segment 3, Segment 4), we provide parameters of the processes we used to generate corresponding segments. We generated segment four in the same way as segment three, but for each component. We added shifts of size 0.3 of the standard deviation of the components for segment 4.

The first change in generating mechanism occurred only in the deterministic components. The second change occurred in model matrix Θ of the VAR(1) process. The third MCGM corresponds to the change in variance–covariance matrix of the VAR(1) process and one component of deterministic process. The fourth change is the change in the mean value for all components. Here, we keep parameters of each component as it is in segment 4 but added the shift 0.3 of standard deviation for segment 4 of the corresponding components.

**Figure 5:**
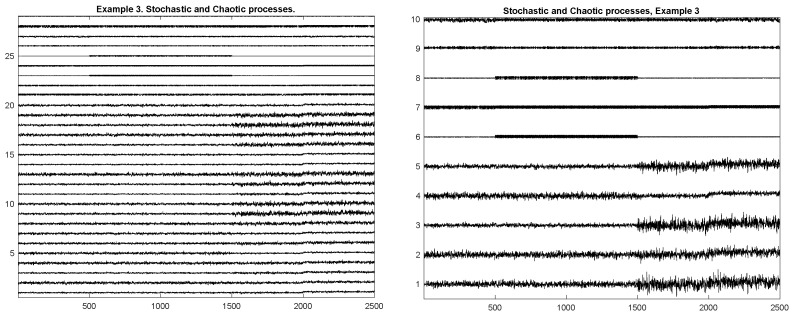
Example 3. Realization of the mixed process (some components are stochastic and some are deterministic). **Left**: All components. **Right**: selected 10 components.

Figure 5 shows an example of the simulated process from Example 3. The Left plot shows all 27 components of the process. In this realization, each homogeneous segment has a length of 500 points. The right plot shows only ten components (five are stochastic and five are deterministic) of the given process, which allows us to see the process’ behavior better.

**Figure 6:**
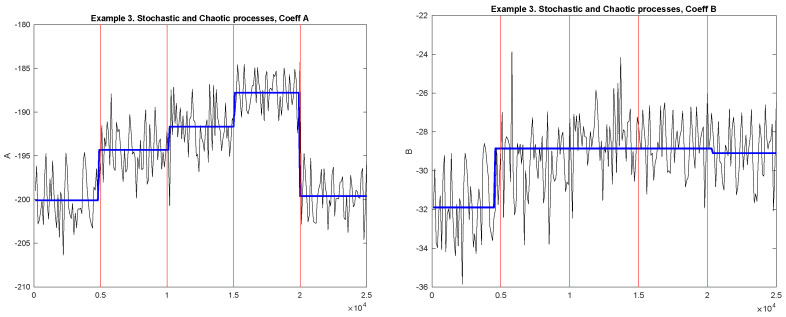
Example 3. Diagnostic sequences and detected MCGM. **Left**: Coefficient A; **Right**: Coefficient B. Black solid lines correspond to the diagnostic sequences; horizontal blue lines correspond to the mean values between the detected change-points. The jump points correspond to the detected change-points. The vertical red lines correspond to the true change-points.

Figure 6 shows the examples of the diagnostic sequences *A* (Left plot) and *B* (Right plot) and detected change-points for Example 3. Black solid lines correspond to the diagnostic sequences; horizontal blue lines correspond to the mean values between the detected change-points. The jump points correspond to the detected change-points. The vertical red lines correspond to the actual change-points.

The numerical results are presented in Table 10 and Table 11. The percentage of the number of detected points for diagnostic sequences of coefficients *A* and *B* in 1000 replications are presented in Table 10. The percentages of correctly found numbers of each of the four change-points (true positive rate) and corresponding bootstrap confidence intervals are presented in Table 11. The results for the old method are presented in Table 12.

In this example, the diagnostic sequence of coefficient *A* works better for this example. Let us also observe that the second change in generating mechanism (change in the parameter α of the Logistic map and parameter *a* of the Hénon map) is better detected by the old method (see, Table 12). For other MCGMs, the new method works better.

## 5. Conclusions

In this paper, we proposed the model-free method for retrospective detection of moments of changes in generating mechanisms of multivariate time series. The detection of moments of changes in the generating mechanism is important for the subsequent analysis of the collected data. It allows one to carry out segmentation of the data on homogeneous fragments.

In econometrics, the moments of changes in the generation mechanism of multidimensional data are called structural breaks. The problem of detection of changes in chaotic processes arises in the study of the phenomenon of multifractality.

However, often, the mechanism for generating a time series is either known inaccurately or entirely unknown. Typical examples here are multidimensional EGG signals, financial time series, some biological data, etc. Thus, it is essential to develop methods for detecting the moments of changes in the generation mechanism of time series that do not use models.

We proposed the method for the detection of changes regardless of the generating mechanisms of arbitrary nature. This method is an extension of our approach proposed in [12]. The given simulation results demonstrate the effectiveness of the new version of our method.

In our simulation study, we considered three examples. In the first example, we simulated the VAR(1) process with four change-points. The first one corresponds to the change of the mean values of 50% of the component. The change was approximately 0.77σ=2, where σ is the maximal standard deviation of the components. In case of change in the mean of all components, we detected a change of 0.77σ=2. The new method was able to catch them while our previous method did not detect this change. The new method detected the change in the variance–covariance matrix *U* but the old approach did not. Both methods were able to detect the change in the model matrix Θ. To measure differences between the matrices and variance–covariance matrices for which we were able to detect changes, we provided the following spectral norms $∥\Theta_{1}-\Theta_{2}{∥}_{2}$ and $∥\Sigma_{u_{1}}-\Sigma_{u_{2}}{∥}_{2}$ and ratios $∥\Theta_{1}-\Theta_{2}{∥}_{2}/(0.5∥\Theta_{1}{∥}_{2}+0.5∥\Theta_{2}{∥}_{2})$, $∥\Sigma_{u_{1}}-\Sigma_{u_{2}}{∥}_{2}/(0.5∥\Sigma_{u_{1}}{∥}_{2}+0.5∥\Sigma_{u_{2}}{∥}_{2})$.

In the second example, we detected changes in multivariate chaotic deterministic processes with some dependent components. In this case, we were able to detect a shift $0.5\sigma_{i}$, where $\sigma_{i}$ is the standard deviation of the *i*-th component. We observed that old and new methods detected changes in the parameters of the chaotic deterministic processes with similar accuracy; however, only the new approach enabled us to detect changes in the mean of multivariate chaotic deterministic processes.

In the last example, we considered the process with stochastic and chaotic components. We observed that the new method was superior to detecting changes in the VAR(1) process and a change in the mean value of the process.

The limitation of our method is that it requires a relatively long sequence of multivariate time series. To calculate the complexity coefficient, we need at least 100 data points. To ensure that the limiting distribution for statistics from our 3-step algorithm will start to work, the diagnostic sequence for each homogeneous increment should be several dozen. In our examples, we used non-overlapping windows. Note that when a non-overlapping window intersects any MCGM, the mathematical expectation of the sequence of complexity coefficient varies according to a certain transient process from one constant to another. However, when the window size is much less than the length of any homogeneity interval, such a transient process does not significantly affect the estimates of MCGM.

A fundamental feature of the proposed method is its independence from the model of the observed process. As far as we know, model-free methods for solving such problems have not been considered in the literature. The independence from the process model is achieved by utilizing our theory of the ϵ-complexity of continuous vector functions, which is consistent with the general idea of A.N. Kolmogorov on how it is expedient to evaluate the complexity of an object.

## Figures and Tables

**Table 1:** Example 1. Description of the segments in simulations.

	Segm 1	Segm 2	Segm 3	Segm 4	Segm 5
Mean	$\mu_{1:50}=0$	$\mu_{1:25}=2$, $\mu_{26:50}=0$,	$\mu_{1:50}=0$	$\mu_{1:50}=0$	$\mu_{1:50}=1.5$
Model Matrix	$\Theta_{1}$	$\Theta_{1}$	$\Theta_{2}$	$\Theta_{2}$	$\Theta_{2}$
Norms of Θ	0.13	0.13	0.09	0.09	0.09
Variance–covariance	$U_{1}$	$U_{1}$	$U_{1}$	$U_{2}$	$U_{2}$
Norms of *U*	44.2	44.2	44.2	52.6	52.6

**Table 2:** Example 1. The percentage of the number of detected change-points for diagnostic sequences of coefficients *A* and *B*.

♯ of Detected Points	Coeff A(t)	Coeff B(t)
1	0%	32.3%
2	0%	65.9%
3	1%	1.6%
4	74.5%	0.2%
5	20%	0%
7	0.8%	0%

**Table 3:** Example 1. The percentages of correctly found numbers of each of the four change-points (true positive rate) and corresponding bootstrap confidence intervals using the proposed method.

Change-Point	True Positive Rate, Coeff *A*	CI, Coeff *A*	True Positive Rate, Coeff *B*	CI, Coeff *B*
1	96.6%	(4800, 5200)	0.1%	N/A
2	98.9%	(9800, 10,176)	26.1%	(9100, 10,900)
3	95.5%	(14,500, 15,300)	12.7	(14,100, 15,733)
4	78.3 %	(19,900, 20,100)	0	N/A

**Table 4:** Example 1. The percentages of correctly found numbers of each of the four change-points (true positive rate) and corresponding bootstrap confidence intervals using the old method.

Change-Point	True Positive Rate, Coeff *A*	CI, Coeff *A*	True Positive Rate, Coeff *B*	CI, Coeff *B*
1	1.6%	(4100, 5800)	2.3%	(4100,5700)
2	99.8%	(9900, 10,200)	89.9%	(9600, 10,750)
3	5.8%	(14,200, 15,900)	3.0	(14,125, 15,875)
4	56.4	(19,300, 20,700)	37.5	(19,300, 20,700)

**Table 5:** Example 2. Processes and changes in the parameters.

Component	Process	Segment 1	Segment 2	Segment 3
1	Logistic map, (6)	α=3.94	α=4	$\alpha_{3}=3.89$
2	Hénon Map, x (10)	$a_{1}=1.5$	$a_{2}=1.3$	$a_{3}=1.4$
		$b_{1}=0.2$	$b_{2}=0.2$	$b_{3}=0.2$
3	Hénon Map, y (10)			
4	Ikeda map, x (11)	$\mu_{1}=0.9$	$\mu_{2}=0.87$	$\mu_{3}=0.9$
		$c_{1}=1.97$	$c_{2}=1.99$	$c_{3}=1.97$
5	Ikeda map, y (11)			
6	Quadratic map (7)	$c_{1}=2$	$c_{2}=1.87$	$c_{3}=1.95$
7		Process 3 (8)	Interval map (9)	Interval map (9)

**Table 6:** Example 2. The percentage of the number of detected change-points for diagnostic sequences of coefficients *A* and *B*.

♯ of Detected Points	Coeff A(t)	Coeff B(t)
1	16%	0%
2	13.7%	99.5%
3	52%	0.5%
4	15.2%	0%
5	1.4%	0%
6	0.3%	0%
7	0.1%	0%

**Table 7:** Example 2. The percentages of correctly found numbers of each of the three change-points (true positive rate) and corresponding bootstrap confidence intervals, proposed method.

Change-Point	True Positive *A*	CI, Coeff *A*	True Positive *B*	CI, Coeff *B*
1	66.9%	(4900, 5000)	98.1%	(4900, 5000)
2	69.4%	(9700, 10,700)	75.1%	(10,000, 10,100)
3	95.3 %	(14,800, 15,800)	0.2%	N/A

**Table 8:** Example 2. The percentages of correctly found numbers of each of the three change-points (true positive rate) and corresponding bootstrap confidence intervals using the old method.

Change-Point	True Positive *A*	CI, Coeff *A*	True Positive *B*	CI, Coeff *B*
1	1.1%	(51,000, 5900)	98.8%	(4900, 5000)
2	5.8%	(9400, 10,100)	74.9%	(10,000, 10,100)
3	0 %	N/A	0.6%	N/A

**Table 9:** Example 3. Processes and changes in the parameters.

Component	Process	Segm 1	Segm 2	Segm 3	Segm 4
1–20	VAR(1)	$\Theta_{1}$, $U_{1}$	$\Theta_{1}$, $U_{1}$	$\Theta_{2}$, $U_{1}$	$\Theta_{2}$, $U_{2}$
	norms Θ	0.16	0.02	0.02	0.16
	norms *U*	8.6	8.6	8.6	12.7
21	Logistic map, (6)	α=4	α=3.98	$\alpha_{3}=3.97$	$\alpha_{4}=3.98$
22	Hénon Map, x (10)	$a_{1}=1.5$	$a_{2}=1.3$	$a_{3}=1.4$	$a_{4}=1.4$
		$b_{1}=0.2$	$b_{2}=0.2$	$b_{3}=0.2$	$b_{4}=0.2$
23	Hénon Map, y (10)				
24	Hénon Map, x (10)	$a_{1}=1.5$	$a_{2}=1.2$	$a_{3}=1.2$	$a_{4}=1.4$
		$b_{1}=0.18$	$b_{2}=0.2$	$b_{3}=0.2$	$b_{4}=0.2$
25	Hénon Map, y (10)				
26	Ikeda map, x (11)	$\mu_{1}=0.9$	$\mu_{2}=0.86$	$\mu_{3}=0.86$	$\mu_{4}=0.86$
		$c_{1}=1.97$	$c_{2}=1.995$	$c_{3}=1.995$	$c_{4}=1.995$
27	Ikeda map, y (11)				
28	Quadratic map (7)	$c_{1}=2$	$c_{2}=1.9$	$c_{3}=1.9$	$c_{4}=1.97$

**Table 10:** Example 3. The percentage of the number of detected change-points for diagnostic sequences of coefficients *A* and *B*.

♯ of Detected Points	Coeff A(t)	Coeff B(t)
1	7.3%	25.1%
2	13.6%	51%
3	60%	19.2%
4	11.6%	3.6%
5	4.6%	0.9%
6	2.2%	0.2%
7	0.7%	0%

**Table 11:** Example 3. The percentages of correctly found numbers of each of the four change-points (true positive rate) and corresponding bootstrap confidence intervals using the proposed method.

Change-Point	True Positive *A*	CI, Coeff *A*	True Positive *B*	CI, Coeff *B*
1	98.8.9%	(4700, 5400)	79.1.1%	(4300, 5770)
2	71.0%	(9600, 10,600)	51.5%	(9100, 10,700)
3	81.2%	(14,400, 15,300)	0.4%	(14,100, 15,900)
4	82.5%	(20,000, 20,100)	0.6%	N/A

**Table 12:** Example 3. The percentages of correctly found numbers of each of the four change-points (true positive rate) and corresponding bootstrap confidence intervals using the old method.

Change-Point	True Positive *A*	CI, Coeff *A*	True Positive *B*	CI, Coeff *B*
1	1.9%	(4200, 5900)	79.2.9%	(4200, 5800)
2	99.6.0%	(9900, 10200)	88.7.5%	(9600, 10,700)
3	5.4.2%	(14,100, 15,820)	3.5%	(14,300, 15,900)
4	58.5.5%	(19,200, 20,700)	45.5.6%	(19,200, 20,800)

## Abbreviations

The following abbreviations are used in this manuscript:

VAR	Vector Autoregressive model
MCGM	Moment of Changes in Generating Mechanism
